# Stem cell-derived extracellular vesicles for myocardial infarction: a meta-analysis of controlled animal studies

**DOI:** 10.18632/aging.101814

**Published:** 2019-02-21

**Authors:** Lihong Yang, Jialu Zhu, Cong Zhang, Juntao Wang, Fengyang Yue, Xingtai Jia, Hongzhi Liu

**Affiliations:** 1 Department of Cardiac Function Evaluation, Henan Provincial People’s Hospital, Zhengzhou, Henan, China; 2 Department of Cardiology, Zhengzhou University People’s Hospital, Zhengzhou, Henan, China; 3 Department of Electrocardiology, Fuwai Central China Cardiovascular Hospital, Zhengzhou, Henan, China; 4 Department of Cardiology, Zhengzhou Central Hospital Affiliated to Zhengzhou University, Zhengzhou, Henan, China; 5 Department of Cardiology, The Second Affiliated Hospital of Zhengzhou University, Zhengzhou, Henan, China; 6 Department of Cardiology, Fuwai Central China Hospital, Zhengzhou, Henan, China; 7 Department of Cardiology, Henan Provincial People’s Hospital, Zhengzhou, Henan, China; * Equal contribution

**Keywords:** extracellular vesicles, myocardial infarction, systematic review, meta-analysis

## Abstract

**Aims:**

Stem cell-derived extracellular vesicles (EVs) have emerged as a promising therapy for myocardial infarction, but its effects remain incompletely understood. We aim to systematically review the efficacy of EVs on myocardial infarction in both small and large animals.

**Methods:**

On April 5, 2018, we searched the PubMed, Embase and Web of Science databases using variations of “myocardial infarction” and “extracellular vesicle”. Controlled studies about the treatment effects of stem cell-derived EVs in myocardial infarction animal model were included. Meta-regression analysis was used to reveal the factors affecting the EVs treatments.

**Results:**

Of 1210 studies retrieved, 24 were eligible for meta-analysis. EVs injection was associated with the improvements of left ventricular ejection fraction (12.65%), fractional shortening (7.54%) and the reduction of infarct size/area at risk (-15.55%). Meta-regression analysis did not reveal the association between treatment efficacy and type of stem cell, ligation-to-injection interval, route of delivery, dosage of delivery or follow-up period (all P values > 0.05). The median quality score of eligible studies was only 1, indicating potential risks of bias.

**Conclusion:**

Stem cell-derived EVs improve cardiac function and reduce infarct size in myocardial infarction animals, but current pool-up study reveals no associations between common factors and treatment effects.

## Introduction

Myocardial infarction (MI) remains an important component of global health loss, although pharmacological treatments and interventional strategies have been greatly developed to reduce the morbidity and mortality in the past decades [1]. One of the main goals of MI management is to salvage and even regenerate the infarcted myocardium. Despite timely coronary interventions and sufficient evidence-based pharmacological treatments, a large proportion of MI patients still suffer from cardiomyocyte apoptosis, ventricular wall thinning, cavity enlargement and finally heart failure [2]. The past decades have seen the surge of many animal studies and clinical trials conducted to reveal the role of stem cells in MI repair, yet controversies remain regarding the mechanisms and efficacy of stem cells for MI [3]. Cell therapy has some inherent disadvantages such as low immediate and long-term cell retention rate after implantation to target tissues and low cell survival rate due to the adverse environments of MI region. Recently, stem cell-free therapies including extracellular vesicles (EVs) have been proposed to repair the infarcted myocardium.

EVs are bilayer lipid-enclosed microvesicles derived from endosomes and secreted by almost all types of cells such as cancer cells, endothelial cells, and stem cells. EVs contain various bioactive components including nucleic acids, proteins, lipids and carbohydrates, functioning as intercellular message carriers under both physiologic and pathologic conditions [4]. Currently, studies have indicated stem cell-derived EVs as a promising therapeutic agent for myocardial infarction, heart failure and dilated cardiomyopathy [5]. Intramyocardial and intravenous injections of EVs have been demonstrated to have anti-apoptotic, anti-fibrotic and angiogenic effects on infarcted myocardium [6–8]. Despite various aims and setups, most animal studies using EVs for MI repair commonly follow four steps: isolation and characterization of EVs, ligation of coronary artery, injection of EVs to infarct zone, and assessments of cardiac function and infarct size. In this study, we quantitatively analyse the treatment effects of stem cell-derived EVs on improving cardiac function and reducing infarct size in animal models after myocardial infarction.

## RESULTS

**Figure 1:**
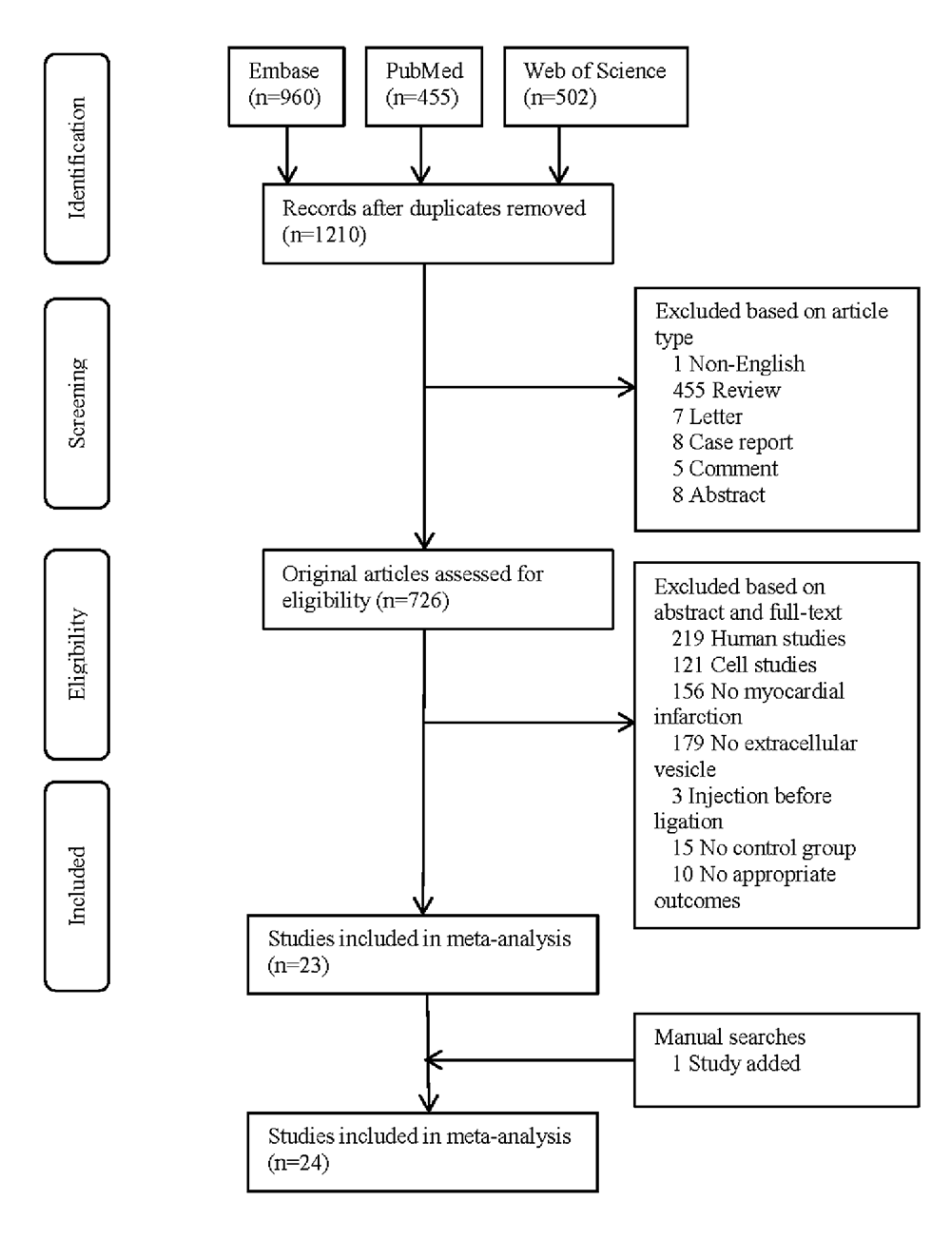
PRISMA flowchart of study selection.

After removal of duplicates, 1210 studies were primarily screened by article type. Then 726 original articles were further screened by abstract and full-text, resulting in 23 eligible studies. One study was added by manually searching the reference lists of eligible articles and review articles [9]. Overall, 24 studies were finally included in statistical analysis (Figure 1).

### Baseline characteristics

The eligible studies contained 31 comparisons and 524 MI animals, including 272 animals in EVs group and 252 animals in control group (Supplement 1). EVs were frequently isolated from mesenchymal stem cells (14/31), cardiac progenitor cells (7/31), and cardiosphere-derived cells (7/31). The size of isolated EVs ranged between 20-1000 nm (mostly 50-200 nm). Surface markers included CD 63, CD 9 and CD 81 were used to identify and sort out EVs from other components. A variety of microRNAs were reported in EVs, such as miR-210 and miR-451. Most studies ligated the left anterior descending artery and injected EVs to the animals intramyocardially (22/31) or intravenously (8/31). The median dosage was 100 µl. The median time from injection to examination was 4 weeks.

### EF

**Figure 2:**
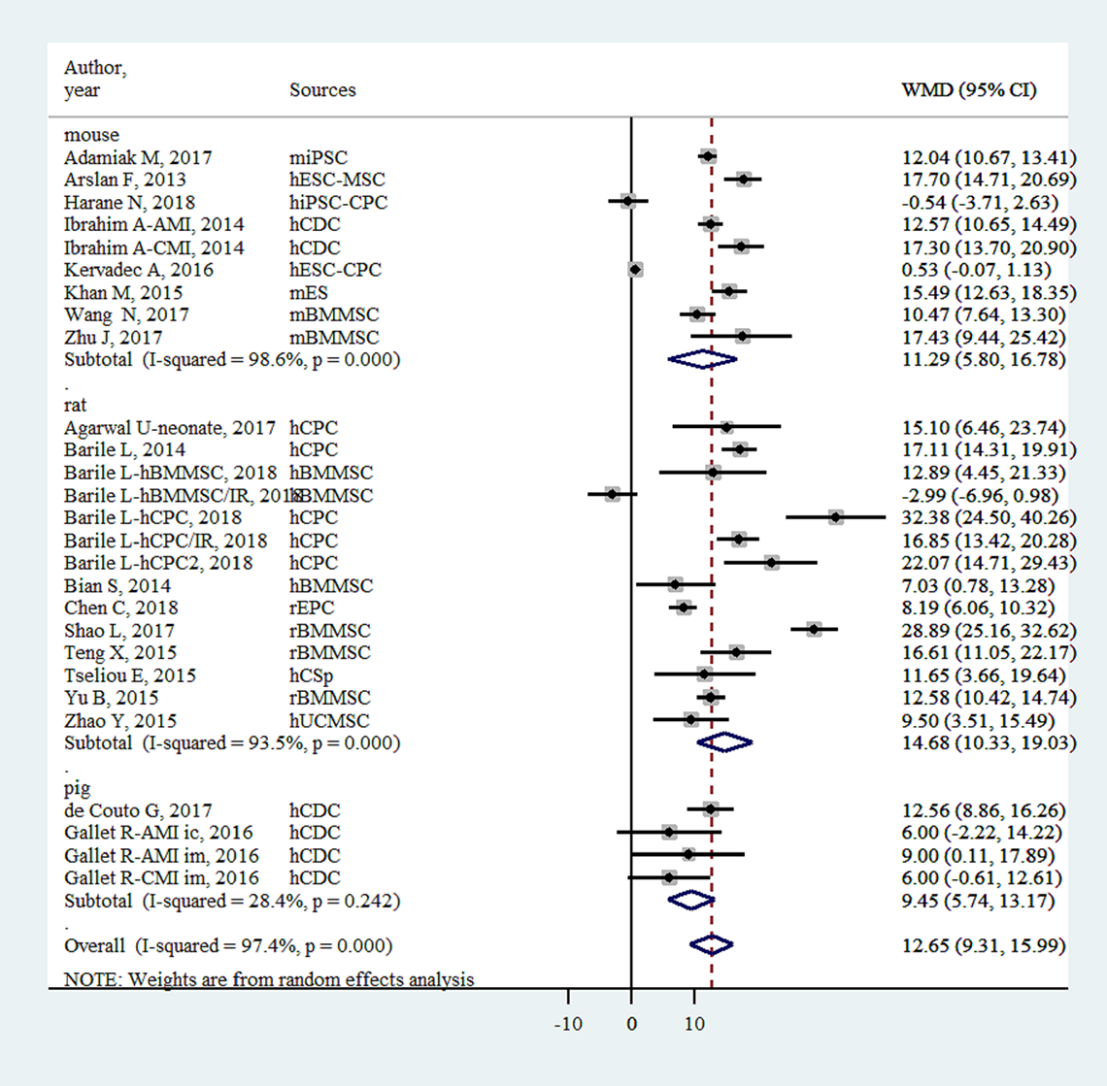
Improvement of left ventricular ejection fraction with injection of stem-cell-derived extracellular vesicles.

EVs injection was associated with an EF improvement of 12.65% (95% confidence interval: 9.31- 15.99%, P<0.001, Figure 2). The improvement was significantly higher in small animals (13.32% [9.66%-16.98%], P < 0.001) than in large animals (9.45% [5.74-13.17%], P < 0.001).

### FS

**Figure 3:**
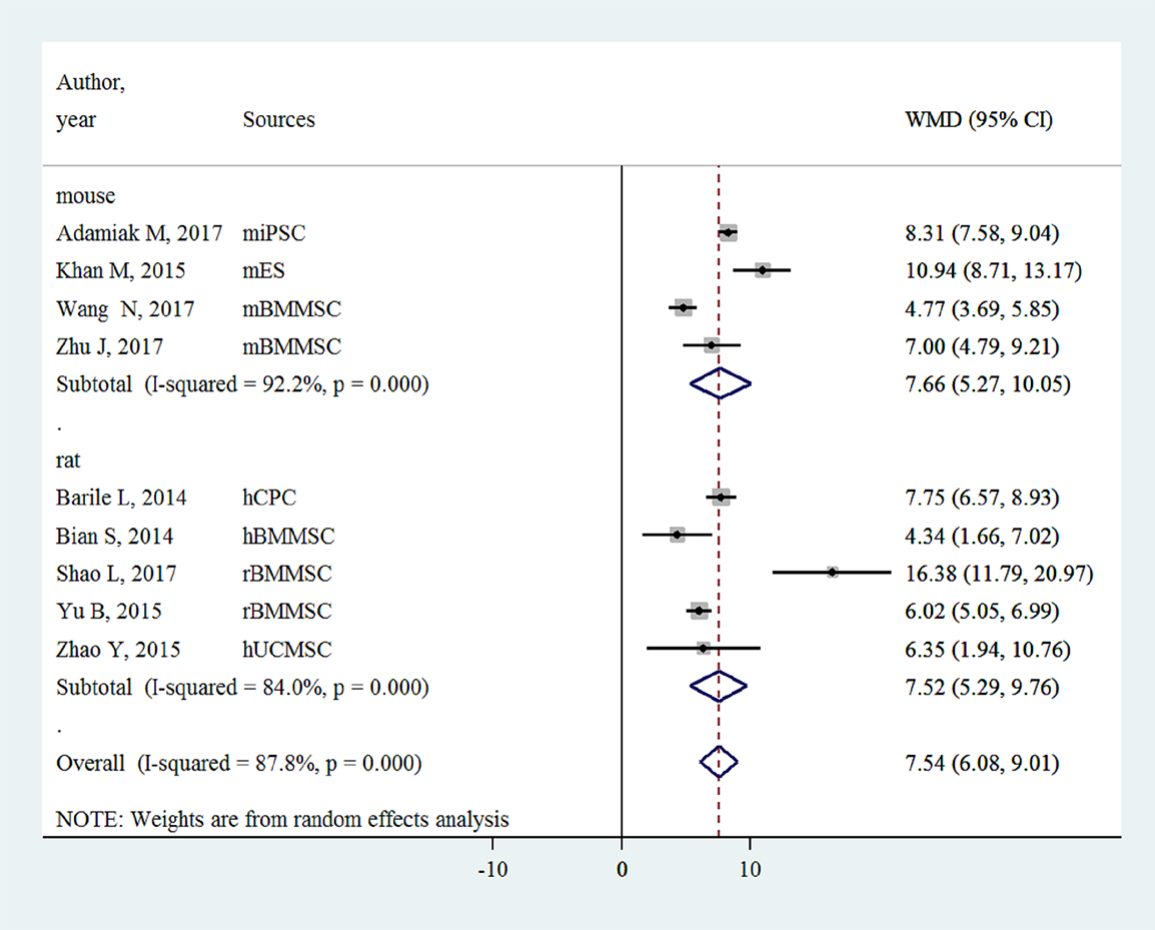
Improvement of left ventricular fractional shortening with injection of stem-cell-derived extracellular vesicles.

FS was only reported in small-animal studies (n = 9). EVs injection was associated with an FS improvement of 7.54% (6.08- 9.01%, P<0.001, Figure 3).

### IS/AAR

**Figure 4:**
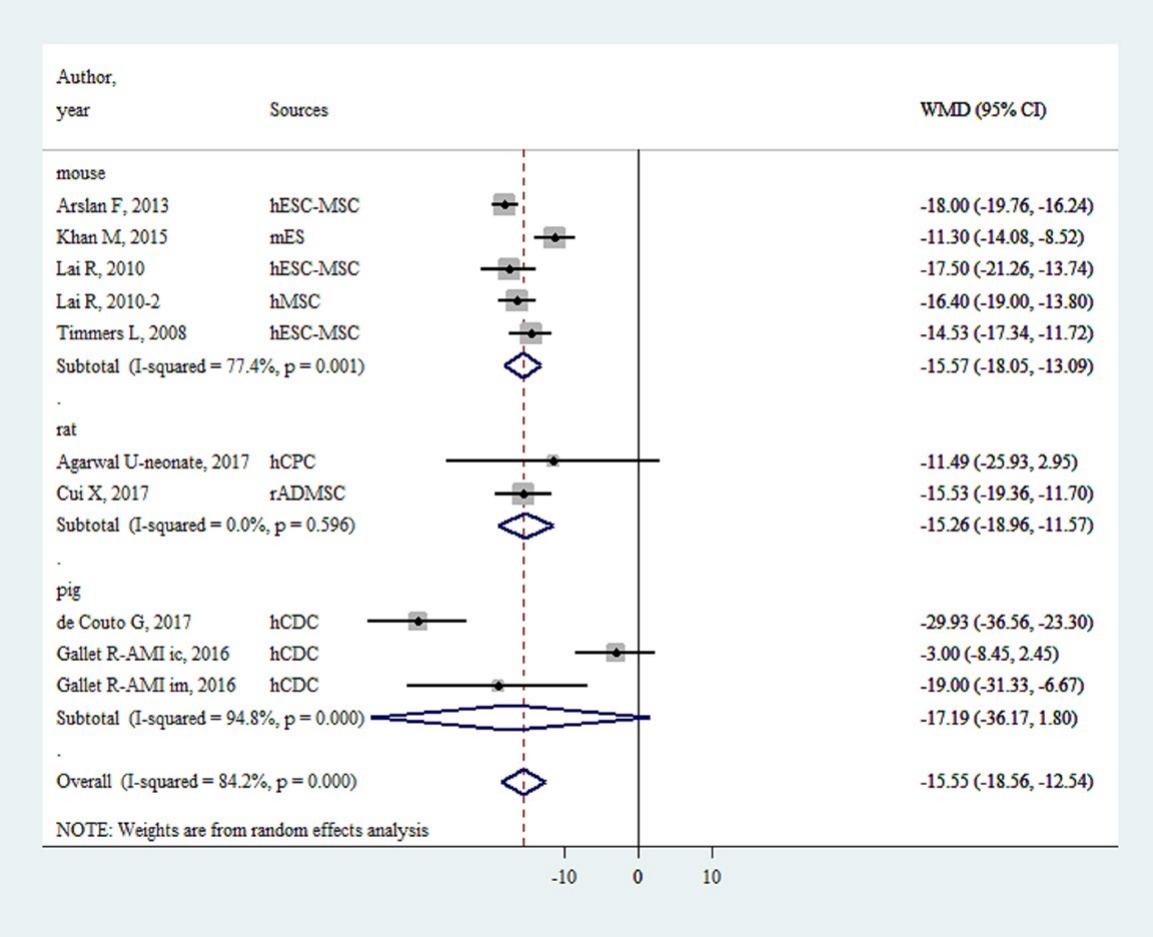
Reduction of infarct size/area at risk with injection of stem-cell-derived extracellular vesicles.

EVs injection was associated with an IS/AAR reduction of -15.55% (-18.56%~ -12.55%, P < 0.001, Figure 4). The effect sizes for large animals and small animals were -17.19% (-36.17%~1.80%, P < 0.001) and -15.50% (-17.57%~ -13.42%, P < 0.001), respectively.

### Meta-regression analysis

Meta-regression analysis did not show significant associations between effect size and type of stem cell, ligation-to-injection interval, route of delivery and follow-up period (Supplement 2, P > 0.05). Dosage was previously reported to be associated with efficacy of EVs injection, but our meta-regression analysis did not show statistical significance (P = 0.34 for all animals, and P = 0.88 for small animals).

### Risk of bias and sensitivity analysis

The median quality score was 1 (Supplement 3). Although random allocation was reported in 10 comparisons, none of them provided sufficient details on the generation procedures of random sequence. Blinding injection was only performed in 3 comparisons. Blinding assessment was conducted in 11 comparisons. Sensitivity analysis was performed by removing the highest and lowest values. Removing the highest value did not result in marked fluctuation of EF (10.90% [7.22-14.58%]), FS (5.19% [2.24-8.15%]) or IS/AAR (-12.84% [-16.76~-8.92%]), compared with the original results. Similarly, removing the highest and lowest values of other outcomes did not result in any significant changes of the original effect sizes.

## DISCUSSION

To the best of our knowledge, this is the first preclinical systematic review and meta-analysis of large- and small- animal experiments to provide evidence summary to support the treatment efficacy of EVs injection for MI. Our major finding is that the injection of stem cell-derived EVs is effective in improving EF and FS and reducing IS/AAR in MI animals, but the overall quality of currently available studies is low and requires more improvements in the future. It is noteworthy that compared with previous preclinical meta-analyses of stem cell therapy, stem-cell-derived EVs injection seems to be more effective than cardiac stem cell injection and mesenchymal stem cell injection in improving cardiac function (improvement of EF: 12.65% *vs*. 10.66% *vs*. 10.79%) in animal models [10,11]. Embryonic stem cell-derived EVs appear to be more effective in improving EF, based on the evidence from small animals. Mouse embryonic stem cell-derived EVs promote endogenous repair mechanisms by augmenting cardiac progenitor cell survival and proliferation, indicating the complicated interactions of exogenous and endogenous stem cells during cardiac remodeling [8]. This kind of interactions among various types of cells may provide synergetic effects to allow better regeneration of infarcted myocardium. Moreover, EVs may also exert beneficial effects on myocardial infarction through the conversion of inactivated cells to activated cells. For instance, CDCs-derived EVs are capable of converting inert cells (e.g., fibroblasts) to therapeutically active cells [6].

As a cell-free therapy, EVs injection does not have the inherent problems of cell therapy such as low cell survival and teratoma formation, thereby serving as an efficient and safe alternative to cell therapy [12]. EVs injection facilitates post-infarct cardiac remodelling by reducing TUNEL-positive area and fibrotic area and promoting angiogenesis [13]. These beneficial effects of EVs have been mainly attributed to the intracellular components, e.g., microRNAs and growth factors. Similarities of intracellular components such as microRNAs (miR-126, miR-146a and miR-210) between EVs and parent cells have been recently demonstrated by a study, which also showed the abundance of some microRNAs specifically in EVs [14]. These special microRNAs are likely to confer better cardioprotective effects to EVs than their parent stem cells. More importantly, exogenous microRNAs and other bioactive molecules may also be delivered by EVs to heal ischemic myocardium. By far, the cardioprotective effects of EVs have been associated with the activation of multiple signalling pathways such as the Wnt/β-catenin signaling pathway, PI3K/Akt pathway and ERK1/2 signaling pathway [7,15,16]. The modification and pretreatment of EVs may further empower them to become a useful tool for cardiac repair after myocardial infarction. EVs secreted from the stem cells with GATA-4 overexpression, Akt overexpression and hypoxia-pretreatment have shown better improvements of cardiac function in animal models, as compared with the EVs derived from unmodified stem cells [17–19]. Although all these methods have been proposed to cultivate the benefits of EVs for post-infarct cardiac repair, currently our understandings of the underlying pathophysiological mechanisms remain incomplete and need further investigations.

Follow-up period is crucial for the beneficial effects of stem cell therapy for animal model of MI, as shown by previous meta-analyses that the treatment effect often drops with time [20,21]. However, our analysis did not reveal the association between follow-up period and treatment effects of EVs injection. This may be due to the fact that in our eligible studies, EVs were derived from various stem cells and injected at different dosages, so the effect of follow-up period on MI might have been somehow offset by these factors. As shown in our meta-analysis, EVs have been isolated and identified using different methods across various studies. Thus, standardization and optimization of isolation and identification techniques serve as the cornerstone for the better translation of EVs from laboratory to clinic. The overall quality of experiment conduction was low in eligible animal studies as reflected by a low SYCLE score of 1. The lack of randomization and blinding in animal studies might have introduced the risks of overestimation of treatment effects of EVs into original studies as well as into our meta-analysis. Considering all these factors which might have affected the results of animal studies to different extents, we believe that our findings of no associations between type of EVs, or the route, dosage and timing of injection have been more likely affected by interstudy heterogeneities than by the real treatment effect of EVs. In the future, *in-vivo* studies may be designed in a more rigorous way and with reference to the requirements of human randomized controlled trials.

### Study limitations

First, although we have shown that stem cell-derived EVs injection could generally improve cardiac function, we did not identify any factors which might affect the efficacy of EVs treatments. More rigorously designed animal studies are in fact needed to reveal the treatment effect of EVs. Second, our meta-analysis has included mostly small animal models (mice and rats, 22/24) which might have been less clinically relevant than large animals (rabbits, dogs and pigs). The inclusion of a large amount of small-animal studies might have also overestimated the treatment effect of stem cell-derived EVs on MI.

## CONCLUSIONS

Stem cell-derived EVs are effective in improving cardiac function and reducing infarct size in animal MI models, but current meta-analysis did not reveal any significant associations between treatment effects and relevant factors such as timing, route and dosage of injection. More rigorously designed animal studies are needed to investigate the treatment effects of EVs.

## MATERIALS AND METHODS

Our study has been registered on the CAMARADES (Collaborative Approach to Meta-Analysis and Review of Animal Data from Experimental Studies) website (http://www.dcn.ed.ac.uk/camarades/research.html#protocols). Study protocol is freely downloadable. We followed a previously published guideline of reporting preclinical systematic review and meta-analysis [22].

### Literature search

On April 5, 2018, we searched the PubMed, Embase and Web of Science using various combinations of “myocardial infarction” and “extracellular vesicle” (Supplement 4). Publication date was from inception to April 5, 2018. Publication language was limited to English. We also examined the reference lists of eligible studies and review articles to identify possible relevant publications.

### Study selection

After removal of duplicates, the studies were screened according to the following inclusion criteria and exclusion criteria. Inclusion criteria: 1) original articles; 2) MI animals; 3) intervention group injected with stem-cell-derived EVs diluted in vehicles; 4) control group injected with vehicles alone (phosphate buffer saline, normal saline or culture medium); 5) reporting of at least one of the following outcomes: left ventricular ejection fraction (EF), left ventricular fractional shortening (FS), or infarct size/area at risk (IS/AAR). Exclusion criteria: 1) article type: review, case report and letter; 2) EVs injected before coronary ligation; 3) control group untreated. Two investigators independently screened the studies. Differences were resolved by consensus.

### Data extraction

Data were extracted from eligible studies to a predesigned electronic table which included publication details, study design, and outcomes. If data were only reported in figures, we would extract the data with a digitalized tool WebPlotDigitizer [23]. The authors of potential eligible studies would also be contacted via email for experimental data if they were not reported in their papers. The paper would be excluded from statistical analysis if the emails were not responded before the initiation of statistical analysis (~ 3 weeks). The data extraction table was completed by two investigators independently and then checked by the third investigator.

### Quality assessment

We assessed the quality of eligible studies using the following three items adapted from the SYRCLE risk of bias tool: random allocation, blinding injection, and blinding evaluation [24]. Each item was scored “Yes” (given 1 point) or “No” (given 0 point). Two investigators independently scored the eligible studies. Differences were resolved by consensus.

### Statistical analysis

In our meta-analysis, small animals were defined as rats and mice, while large animals as pigs. Weighted mean difference (WMD) and the corresponding 95% confidence interval (CI) were calculated for each comparison of studies. DerSimonian-Laird random-effects model was used to pool up the extracted data because we expected a huge heterogeneity across different eligible studies. Q statistic and I^2^ statistic were used to quantify the interstudy heterogeneity. Meta-regression analysis was performed to identify how factors including type of stem cell, ligation-to-injection interval, route of delivery, dosage of delivery and follow-up period affected the treatment effects of stem cell-derived EVs. Sensitivity analysis was conducted by removing extreme values and recalculating the effect size and 95% confidence interval. The difference was compared between before and after the removal of extreme values.

## SUPPLEMENTARY MATERIAL

Supplements 1-4
