# Supplementary material for: Stem cell-derived extracellular vesicles for myocardial infarction: a meta-analysis of controlled animal studies
Source: Aging (Albany NY). 2019 Feb 21;11(4):1129–50. doi: 10.18632/aging.101814 (PMC6402509; doi:10.18632/aging.101814)
Supplement: Supplements 1-4 [file aging-11-101814-s001.pdf]

SUPPLEMENTARY MATERIAL

Supplement 1. Baseline characteristics of eligible studies.
Supplement 2. Meta-regression analysis.
Supplement 3. Quality of eligible studies.
Supplement 4. Search strategies.
Supplementary References

Supplement 1. Baseline characteristics of eligible studies.

Author, year	class	Species	Strain	Sex, age, weight	Ligation	Reperfusion	Control		L-to-I time (min)	Route	Total dose (µl)	Follow-up	Imaging	N	EF (%)		Con.		FS (%)		Con.		IS/AAR (%)		Con.	
de Couto G, 2017 [1]	CDC	pig	Yucatan	F, adult, 80 kg	LAD	1	IMDM	2	120	im		48 h	ventriculography	9	56.49	2.31	43.93	3.34					51.56	6.53	81.49	1.98
Gallet R-AMI ic, 2016 [2]	CDC	pig	Yucatan	F, adult, 80 kg	LAD	1	IMDM	2	120	ic	10000	48 h	ventriculography	13	48	6	42	9					77	5	80	5
Gallet R-AMI im, 2016 [2]	CDC	pig	Yucatan	F, adult, 80 kg	LAD	1	IMDM	2	120	im	2000	48 h	ventriculography	11	51	6	42	9					61	12	80	5
Gallet R-CMI im, 2016 [2]	CDC	pig	Yucatan	F, adult, 80 kg	LAD	1	IMDM	2	4 w	im	2000	1 m	ventriculography	11	40	4	34	7								
Ibrahim A-AMI, 2014 [3]	CDC	mouse	SCID	M, 3 m	LAD	0	IMDM	2	0	im	80	4 w	echo	16	44	1.57	31.43	2.28								
Ibrahim A-CMI, 2014 [3]	CDC	mouse	SCID	M, 3 m	LAD	0	IMDM	2	3 w	im	80	3 w	echo	12	42.75	3.6	25.45	2.7								
Tseliou E, 2015 [4]	CDC	rat	Wistar	F, 5-6 w	LAD	0	PBS	1	4 w	im	120	4 w	echo	14	44.07	10.82	32.42	3.44								
Agarwal U-neonate, 2017 [5]	CPC	rat	Crl:NIH-Foxn1rnu	250 g	LAD	1	saline	1	30	im	100	25 d	echo	8	73.26	4.76	58.16	7.42					45.56	7.43	57.05	12.7
Barile L, 2014 [6]	CPC	rat	Wistar	M, 250-300 g	LAD	0	PBS	1	60	im	150	5 d	echo	19	64.13	2.95	47.02	3.25	28	1.33	20.25	1.3				
Barile L-hCPC, 2018 [7]	CPC	rat	Wistar	250-300 g	LAD	0	PBS	1	60	im	100	4 w	echo	20	81.54	1.61	49.16	8.94								
Barile L-hCPC/IR, 2018 [7]	CPC	rat	Wistar	250-300 g	LAD	1	PBS	1	30	im	100	4 w	echo	11	77.16	2.19	60.31	3.36								
Barile L-hCPC2, 2018 [7]	CPC	rat	Wistar	250-300 g	LAD	0	PBS	1	60	im	100	4 w	echo	14	68.1	2.06	46.03	8.25								

Author, year	class	Species	Strain	Sex, age, weight	Ligation	Reperfusion	Control		L-to-I time (min)	Route	Total dose (μl)	Follow-up	Imaging	N	EF (%)		Con.		FS (%)		Con.		IS/AAR (%)		Con.	
Harane N, 2018 [8]	CPC	mouse	199 nude		LAD	0	PBS	1	3 w	im	40	7 w	echo	36	41.28	4.77	41.82	4.9								
Kervadec A, 2016 [9]	CPC	mouse	Rj/NMRI-Foxn1nu/Foxn1nu	M, 8 w	LAD	0	alpha-MEM	2	3 w	im	30	6 w	echo	28	2.83	0.84	2.3	0.77								
Chen C, 2018 [10]	EPC	rat	Wistar	M	LAD	0	PBS	1	0	im	100	4 w	ventriculograhy	19	39.34	2	31.15	2.72								
Khan M, 2015 [11]	ES	mouse	C57BL/7	M, 8-12w	LAD	0	PBS	1	0	im	20	8 w	echo	12	43.42	2.53	27.93	2.52	28.74	1.32	17.8	2.45	20.8	2.6	32.1	2.3
Adamiak M, 2017 [12]	iPSC	mouse	C57BL6/J	M, 23-29 g, 11-13 w	LAD	1	PBS	1	48 h	im	150	33 d	echo	21	49.66	1.46	37.62	1.67	29.97	0.92	21.66	0.78				
Arslan F, 2013 [13]	MSC	mouse	C57BL6/J	M,10-12 w, 25-30 g	LCA	1	saline	1	25	iv		4 w	MRI	20	53.83	3.6	36.13	3.21					21	2.2	39	1.8
Barile L-hBMMSC, 2018 [7]	MSC	rat	Wistar	250-300 g	LAD	0	PBS	1	60	im	100	4 w	echo	13	62.05	4.54	49.16	8.94								
Barile L-hBMMSC/IR, 2018 [7]	MSC	rat	Wistar	250-300 g	LAD	1	PBS	1	30	im	100	4 w	echo	10	57.32	3.03	60.31	3.36								
Bian S, 2014 [14]	MSC	rat	Wistar	M, adult, 200 g	LAD	0	PBS	1	30	iv	80	4 w	echo	20	55.44	7.5	48.41	6.73	25.76	3.19	21.42	2.93				
Cui X, 2017 [15]	MSC	rat	SD	M, 275-300 g	LAD	1	PBS	1	35	iv	200	3 h		20									21.61	3.54	37.14	5.06
Lai R, 2010 [16]	MSC	mouse	C57BL16/J	M,10-12 w, 25-30 g	LCA	1	saline	1	25	iv	200	24 h	echo	15									17	3.6	34.5	3.3
Lai R, 2010-2]17]	MSC	mouse	C57BL16/J	M,10-12 w, 25-30 g	LCA	1	saline	1	25	iv	200	24 h	echo	16									18.1	2	34.5	3.3

Author, year	class	Species	Strain	Sex, age, weight	Ligation	Reperfusion	Control		L-to-I time (min)	Route	Total dose (μl)	Follow-up	Imaging	N	EF (%)		Con.		FS (%)		Con.		IS/AAR (%)		Con.	
Shao L, 2017 [18]	MSC	rat	SD	M, 260-280 g	LAD	0	PBS	1	0	im	40	7 d	echo	20	59.66	4.38	30.77	4.13	31.31	7.03	14.93	2.31				
Teng X, 2015 [19]	MSC	rat	SD	M, 250-300 g	LAD	0	PBS	1	60	im	100	4 w	echo	12	79.24	5.08	62.63	4.74								
Timmers L, 2008 [20]	MSC	mouse	BALB/c	10-12 w, 25-30 g	LCA	1	saline	1	25	iv	20	24h		18									20.16	2.91	34.69	3.16
Wang N, 2017 [21]	MSC	mouse	C57BL/6J	M, 8 w	LAD	0	PBS	1	1	iv		4 w	echo	12	50.35	2.93	39.88	1.99	20.47	1.26	15.7	0.5				
Yu B, 2015 [22]	MSC	rat	SD	F, 2-3 m	LAD	0	saline	1	0	im	50	4 w	echo	26	47.71	3.85	35.13	1.56	20.7	1.83	14.68	0.47				
Zhao Y, 2015 [23]	MSC	rat	SD	M, 220-250 g	LAD	0	PBS	1	0	iv	200	4 w	echo	12	59.97	4.58	50.47	5.92	32.76	3.85	26.41	3.94				
Zhu J, 2017 [24]	MSC	mouse	C57BL/6, 20-25 g	M, 6-8 w	LAD	0	PBS	1	30	im	30	4 w	echo	36	38.19	17.2	20.76	5.39	18.95	3.95	11.95	2.8				

Supplement 2. Meta-regression analysis.

EF

Variable	Coefficient	LCI	UCI	P value
class	1.137701	-1.264895	3.540297	0.339
randomization	-5.218478	-12.01666	1.579708	0.126
blinding injection	.859003	-9.458939	11.17695	0.865
blinding assessment	-1.892155	-8.400234	4.615924	0.555
quality score	-1.877796	-5.479191	1.723599	0.293
species	-4.700754	-13.83159	4.430085	0.299
ligation-to-injection time	-3.227312	-7.725924	1.2713	0.152
route	-1.139511	-5.523777	3.244754	0.597
follow up	-1.352479	-3.148747	.4437895	0.134

FS

Variable	Coefficient	LCI	UCI	P value
class	1.004528	-2.38594	4.394996	0.506
randomization	-.8295799	-9.834547	8.175387	0.834
blinding injection	-3.751959	-12.2494	4.745479	0.331
blinding assessment	-.1332895	-5.896159	5.62958	0.958
quality score	-.7855705	-4.346688	2.775547	0.618
species	-	-	-	-
ligation-to-injection time	-1.490393	-7.062023	4.081236	0.547
route	-1.877727	-4.272445	.5169902	0.106
follow up	-.1646326	-1.630776	1.30151	0.798

IS/AAR

Variable	Coefficient	LCI	UCI	P value
class	1.177793	-2.966831	5.322416	0.531
randomization	5.755156	-3.527084	15.0374	0.191
blinding injection	-.4579897	-14.26349	13.34751	0.941
blinding assessment	.3146597	-11.19345	11.82277	0.951
quality score	1.631681	-3.803592	7.066955	0.508
species	-1.439353	-13.26043	10.38172	0.786
ligation-to-injection time	-4.895186	-20.59604	10.80567	0.493
route	.487758	-5.188615	6.164131	0.848
follow up	.6046283	-1.570909	2.780166	0.540

Supplement 3. Quality of eligible studies.

Author, year	Randomization	Blinding injection	Blinding assessment	Total score
de Couto G, 2017 [1]	0	0	0	0
Gallet R-AMI ic, 2016 [2]	1	0	0	1
Gallet R-AMI im, 2016 [2]	1	0	0	1
Gallet R-CMI im, 2016 [2]	1	0	0	1
Ibrahim A-AMI, 2014 [3]	0	0	0	0
Ibrahim A-CMI, 2014 [3]	0	0	0	0
Tseliou E, 2015 [4]	1	0	0	1
Agarwal U-neonate, 2017 [5]	1	1	1	3
Barile L, 2014 [6]	0	0	0	0
Barile L-hCPC, 2018 [7]	0	0	0	0
Barile L-hCPC/IR, 2018 [7]	0	0	0	0
Barile L-hCPC2, 2018 [7]	0	0	0	0
Harane N, 2018 [8]	0	0	1	1
Kervadec A, 2016 [9]	1	0	1	2
Chen C, 2018 [10]	1	0	1	2
Khan M, 2015 [11]	0	0	0	0
Adamiak M, 2017 [12]	0	0	1	1
Arslan F, 2013 [13]	0	1	1	2
Barile L-hBMMSC, 2018 [7]	0	0	0	0
Barile L-hBMMSC/IR, 2018 [7]	0	0	0	0
Bian S, 2014 [14]	0	1	1	2
Cui X, 2017 [15]	1	0	0	1
Lai R, 2010 [16]	0	0	0	0
Lai R, 2010-2 [17]	0	0	0	0
Shao L, 2017 [18]	0	0	1	1
Teng X, 2015 [19]	0	0	0	0
Timmers L, 2008 [20]	1	0	1	2
Wang N, 2017 [21]	0	0	1	1
Yu B, 2015 [22]	0	0	0	0
Zhao Y, 2015 [23]	0	0	0	0
Zhu J, 2017 [24]	1	0	1	2

Supplement 4. Search strategies.

Search strategies were developed with references to a number of articles, especially review articles [25-29]. It's noteworthy that the nomenclature of EVs has been evolving with time. Currently, a variety of names have been adopted by the researchers of different fields around the world. On April 5, 2018, we searched the PubMed, Embase and Web of Science databases with the following strategies:

PubMed (455):
((((vesicle OR vesicles OR microvesicle OR microvesicles OR micro-vesicle OR micro-vesicles OR nanovesicle OR nanovesicles OR nano-vesicle OR nano-vesicles OR microparticle OR microparticles OR micro-particle OR micro-particles OR exovesicle OR exovesicles OR "platelet dust" OR "platelet dusts" OR "apoptotic body" OR "apoptotic bodies" OR exosome OR exosomes OR secretome OR secretomes OR dexosome OR dexosomes OR texosome OR texosomes OR epididymosome OR epididymosomes OR tolerosome OR tolerosomes OR prostasome OR prostasomes OR ectosome OR ectosomes OR "extracellular vesicles"[MeSH Terms] OR "cell-derived microparticles"[MeSH Terms] OR "exosomes"[MeSH Terms])) AND ("myocardial infarction" OR "myocardial infarctions" OR "myocardial infarct" OR "myocardial infarcts" OR "cardiac infarction" OR "cardiac infarctions" OR "ventricular infarction" OR "ventricular infarctions" OR "myocardium infarction" OR "heart infarction" OR "heart infarctions" OR "myocardial ischemia" OR "myocardial ischemias" OR "myocardial necrosis" OR "myocardial necroses" OR "myocardial reperfusion" OR "myocardial reperfusions" OR "myocardial remodel" OR "myocardial remodeling" OR "ventricular remodeling" OR "atrial remodeling" OR "heart attack" OR "heart attacks" OR "coronary occlusion" OR "coronary occlusions" OR "coronary ligation" OR "coronary ligations" OR "myocardial infarction"[MeSH Terms] OR "myocardial ischemia"[MeSH Terms] OR "myocardial reperfusion"[MeSH Terms] OR "ventricular remodeling"[MeSH Terms] OR "atrial remodeling"[MeSH Terms] OR "coronary occlusion"[MeSH Terms])) AND ((("animal experimentation"[MeSH Terms] OR "models, animal"[MeSH Terms] OR "invertebrates"[MeSH Terms] OR "Animals"[Mesh:noexp] OR "animal population groups"[MeSH Terms] OR "chordata"[MeSH Terms:noexp] OR "chordata, nonvertebrate"[MeSH Terms] OR "vertebrates"[MeSH Terms:noexp] OR "amphibians"[MeSH Terms] OR "birds"[MeSH Terms] OR "fishes"[MeSH Terms] OR "reptiles"[MeSH Terms] OR "mammals"[MeSH Terms:noexp] OR "primates"[MeSH Terms:noexp] OR "artiodactyla"[MeSH Terms] OR "carnivora"[MeSH Terms] OR "cetacea"[MeSH Terms] OR "chiroptera"[MeSH Terms] OR "elephants"[MeSH Terms] OR "hyraxes"[MeSH Terms] OR "insectivora"[MeSH Terms] OR "lagomorpha"[MeSH Terms] OR "marsupialia"[MeSH Terms] OR "monotremata"[MeSH Terms] OR "perissodactyla"[MeSH Terms] OR "rodentia"[MeSH Terms] OR "scandentia"[MeSH Terms] OR "sirenia"[MeSH Terms] OR "xenarthra"[MeSH Terms] OR "haplorhini"[MeSH Terms:noexp] OR "strepsirhini"[MeSH Terms] OR "platyrrhini"[MeSH Terms] OR "tarsii"[MeSH Terms] OR "catarrhini"[MeSH Terms:noexp] OR "cercopithecidae"[MeSH Terms] OR "hylobatidae"[MeSH Terms] OR "hominidae"[MeSH Terms:noexp] OR "gorilla gorilla"[MeSH Terms] OR "pan paniscus"[MeSH Terms] OR "pan troglodytes"[MeSH Terms] OR "pongo pygmaeus"[MeSH Terms]) OR ((animals[tiab] OR animal[tiab] OR mice[Tiab] OR mus[Tiab] OR mouse[Tiab] OR murine[Tiab] OR woodmouse[tiab] OR rats[Tiab] OR rat[Tiab] OR murinae[Tiab] OR muridae[Tiab] OR cottonrat[tiab] OR cottonrats[tiab] OR hamster[tiab] OR hamsters[tiab] OR cricetinae[tiab] OR rodentia[Tiab] OR rodent[Tiab] OR rodents[Tiab] OR pigs[Tiab] OR pig[Tiab] OR swine[tiab] OR swines[tiab] OR piglets[tiab] OR piglet[tiab] OR boar[tiab] OR boars[tiab] OR "sus scrofa"[tiab] OR ferrets[tiab] OR ferret[tiab] OR polecat[tiab] OR polecats[tiab] OR "mustela putorius"[tiab] OR "guinea pigs"[Tiab] OR "guinea pig"[Tiab] OR cavia[Tiab] OR callithrix[Tiab] OR marmoset[Tiab] OR marmosets[Tiab] OR cebuella[Tiab] OR hapale[Tiab] OR octodon[Tiab] OR chinchilla[Tiab] OR chinchillas[Tiab] OR gerbillinae[Tiab] OR gerbil[Tiab] OR gerbils[Tiab] OR jird[Tiab] OR jirds[Tiab] OR merione[Tiab] OR meriones[Tiab] OR rabbits[Tiab] OR rabbit[Tiab] OR hares[Tiab] OR hare[Tiab] OR diptera[Tiab] OR flies[Tiab] OR fly[Tiab] OR dipteral[Tiab] OR drosphila[Tiab] OR drosophilidae[Tiab] OR cats[Tiab] OR cat[Tiab] OR carus[Tiab] OR felis[Tiab] OR nematoda[Tiab] OR nematode[Tiab] OR nematoda[Tiab] OR nematode[Tiab] OR nematodes[Tiab] OR sipunculida[Tiab] OR dogs[Tiab] OR dog[Tiab] OR canine[Tiab] OR canines[Tiab] OR canis[Tiab] OR sheep[Tiab] OR sheeps[Tiab] OR mouflon[Tiab] OR mouflons[Tiab] OR ovis[Tiab] OR goats[Tiab] OR goat[Tiab] OR capra[Tiab] OR capras[Tiab] OR rupicapra[Tiab] OR chamois[Tiab] OR haplorhini[Tiab] OR monkey[Tiab] OR monkeys[Tiab] OR anthropoidea[Tiab] OR anthropoids[Tiab] OR saguinus[Tiab] OR tamarin[Tiab] OR tamarins[Tiab] OR leontopithecus[Tiab] OR hominidae[Tiab] OR ape[Tiab] OR apes[Tiab] OR pan[Tiab] OR paniscus[Tiab] OR "pan paniscus"[Tiab] OR bonobo[Tiab] OR bonobos[Tiab] OR troglodytes[Tiab] OR "pan troglodytes"[Tiab] OR gibbon[Tiab] OR gibbons[Tiab] OR siamang[Tiab] OR siamangs[Tiab] OR nomascus[Tiab] OR symphalangus[Tiab] OR chimpanzee[Tiab] OR chimpanzees[Tiab] OR prosimians[Tiab] OR "bush baby"[Tiab] OR prosimian[Tiab] OR bush babies[Tiab] OR galagos[Tiab] OR galago[Tiab] OR pongidae[Tiab] OR gorilla[Tiab] OR gorillas[Tiab] OR pongo[Tiab] OR pygmaeus[Tiab] OR "pongo pygmaeus"[Tiab] OR orangutans[Tiab] OR pygmaeus[Tiab] OR lemur[Tiab] OR lemurs[Tiab] OR lemuridae[Tiab] OR horse[Tiab] OR horses[Tiab] OR pongo[Tiab] OR equus[Tiab] OR cow[Tiab] OR calf[Tiab] OR bull[Tiab] OR chicken[Tiab] OR chickens[Tiab] OR gallus[Tiab] OR quail[Tiab] OR bird[Tiab] OR birds[Tiab] OR quails[Tiab] OR poultry[Tiab] OR poultries[Tiab] OR fowl[Tiab] OR fowls[Tiab] OR reptile[Tiab] OR reptilia[Tiab] OR reptiles[Tiab] OR snakes[Tiab] OR snake[Tiab] OR lizard[Tiab] OR lizards[Tiab] OR alligator[Tiab] OR alligators[Tiab] OR crocodile[Tiab] OR crocodiles[Tiab] OR turtle[Tiab] OR turtles[Tiab] OR amphibian[Tiab] OR amphibians[Tiab] OR amphibia[Tiab] OR frog[Tiab] OR

frogs[Tiab] OR bombina[Tiab] OR salientia[Tiab] OR toad[Tiab] OR toads[Tiab] OR "epidalea calamita"[Tiab] OR salamander[Tiab] OR salamanders[Tiab] OR eel[Tiab] OR eels[Tiab] OR fish[Tiab] OR fishes[Tiab] OR pisces[Tiab] OR catfish[Tiab] OR catfishes[Tiab] OR siluriformes[Tiab] OR arius[Tiab] OR heteropneustes[Tiab] OR sheatfish[Tiab] OR perch[Tiab] OR perches[Tiab] OR percidae[Tiab] OR perca[Tiab] OR trout[Tiab] OR trouts[Tiab] OR char[Tiab] OR chars[Tiab] OR salvelinus[Tiab] OR "fathead minnow"[Tiab] OR minnow[Tiab] OR cyprinidae[Tiab] OR carps[Tiab] OR carp[Tiab] OR zebrafish[Tiab] OR zebrafishes[Tiab] OR goldfish[Tiab] OR goldfishes[Tiab] OR guppy[Tiab] OR guppies[Tiab] OR chub[Tiab] OR chubs[Tiab] OR tinca[Tiab] OR barbels[Tiab] OR barbus[Tiab] OR pimephales[Tiab] OR promelas[Tiab] OR "poecilia reticulata"[Tiab] OR mullet[Tiab] OR mullets[Tiab] OR seahorse[Tiab] OR seahorses[Tiab] OR mugil curema[Tiab] OR atlantic cod[Tiab] OR shark[Tiab] OR sharks[Tiab] OR catshark[Tiab] OR anguilla[Tiab] OR salmonid[Tiab] OR salmonids[Tiab] OR whitefish[Tiab] OR whitefishes[Tiab] OR salmon[Tiab] OR salmons[Tiab] OR sole[Tiab] OR solea[Tiab] OR "sea lamprey"[Tiab] OR lamprey[Tiab] OR lampreys[Tiab] OR pumpkinseed[Tiab] OR sunfish[Tiab] OR sunfishes[Tiab] OR tilapia[Tiab] OR tilapias[Tiab] OR turbot[Tiab] OR turbots[Tiab] OR flatfish[Tiab] OR flatfishes[Tiab] OR sciuridae[Tiab] OR squirrel[Tiab] OR squirrels[Tiab] OR chipmunk[Tiab] OR chipmunks[Tiab] OR suslik[Tiab] OR susliks[Tiab] OR vole[Tiab] OR voles[Tiab] OR lemming[Tiab] OR lemmings[Tiab] OR muskrat[Tiab] OR muskrats[Tiab] OR lemmus[Tiab] OR otter[Tiab] OR otters[Tiab] OR marten[Tiab] OR martens[Tiab] OR martes[Tiab] OR weasel[Tiab] OR badger[Tiab] OR badgers[Tiab] OR ermine[Tiab] OR mink[Tiab] OR minks[Tiab] OR sable[Tiab] OR sables[Tiab] OR gulo[Tiab] OR gulos[Tiab] OR wolverine[Tiab] OR wolverines[Tiab] OR minks[Tiab] OR mustela[Tiab] OR llama[Tiab] OR llamas[Tiab] OR alpaca[Tiab] OR alpacas[Tiab] OR camelid[Tiab] OR camelids[Tiab] OR guanaco[Tiab] OR guanacos[Tiab] OR chiroptera[Tiab] OR chiropteras[Tiab] OR bat[Tiab] OR bats[Tiab] OR fox[Tiab] OR foxes[Tiab] OR iguana[Tiab] OR iguanas[Tiab] OR xenopus laevis[Tiab] OR parakeet[Tiab] OR parakeets[Tiab] OR parrot[Tiab] OR parrots[Tiab] OR donkey[Tiab] OR donkeys[Tiab] OR mule[Tiab] OR mules[Tiab] OR zebra[Tiab] OR zebras[Tiab] OR shrew[Tiab] OR shrews[Tiab] OR bison[Tiab] OR bisons[Tiab] OR buffalo[Tiab] OR buffaloes[Tiab] OR deer[Tiab] OR deers[Tiab] OR bear[Tiab] OR bears[Tiab] OR panda[Tiab] OR pandas[Tiab] OR "wild hog"[Tiab] OR "wild boar"[Tiab] OR fitchew[Tiab] OR fitch[Tiab] OR beaver[Tiab] OR beavers[Tiab] OR jerboa[Tiab] OR jerboas[Tiab] OR capybara[Tiab] OR capybaras[Tiab]) NOT medline[subset])))))
**Embase (960):**<br>**#3 (960)**<br>#1 NOT #2<br>**#2 (402)**<br>#1 AND [embase]/lim NOT [medline]/lim AND 'conference abstract'/it<br>**#1 (1362)**<br>(vesicle OR vesicles OR microvesicle OR microvesicles OR 'micro vesicle' OR 'micro vesicles' OR nanovesicle OR nanovesicles OR 'nano vesicle' OR 'nano vesicles' OR microparticle OR microparticles OR 'micro particle' OR 'micro particles' OR exovesicle OR exovesicles OR 'platelet dust' OR 'platelet dusts' OR 'apoptotic body' OR 'apoptotic bodies' OR exosome OR exosomes OR secretome OR secretomes OR dexosome OR dexosomes OR texosome OR texosomes OR epididymosome OR epididymosomes OR tolerosome OR tolerosomes OR prostasome OR prostasomes OR ectosome OR ectosomes OR 'exosomes'/exp) AND ('myocardial infarction' OR 'myocardial infarctions' OR 'myocardial infarct' OR 'myocardial infarcts' OR 'cardiac infarction' OR 'cardiac infarctions' OR 'ventricular infarction' OR 'ventricular infarctions' OR 'myocardium infarction' OR 'heart infarction' OR 'heart infarctions' OR 'myocardial ischemia' OR 'myocardial ischemias' OR 'myocardial necrosis' OR 'myocardial necroses' OR 'myocardial reperfusion' OR 'myocardial reperfusions' OR 'myocardial remodel' OR 'myocardial remodeling' OR 'ventricular remodeling' OR 'atrial remodeling' OR 'heart attack' OR 'heart attacks' OR 'coronary occlusion' OR 'coronary occlusions' OR 'coronary ligation' OR 'coronary ligations' OR 'heart infarction'/exp OR 'heart muscle ischemia'/exp OR 'heart muscle reperfusion'/exp OR 'heart ventricle remodeling'/exp OR 'coronary artery occlusion'/exp) AND ('animal experiment'/exp OR 'animal model'/exp OR 'invertebrate'/exp OR 'animal'/exp OR 'chordata'/exp OR 'invertebrate chordata'/exp OR 'vertebrate'/exp OR 'amphibian'/exp OR 'bird'/exp OR 'fish'/exp OR 'reptile'/exp OR 'mammal'/exp OR 'primate'/exp OR 'artiodactyla'/exp OR 'carnivora'/exp OR 'cetacea'/exp OR 'chiroptera'/exp OR 'elephant'/exp OR 'hyraxe' OR 'insectivora'/exp OR 'lagomorpha'/exp OR 'marsupialia'/exp OR 'monotremata'/exp OR 'perissodactyla'/exp OR 'rodentia'/exp OR 'scandentia'/exp OR 'sirenia'/exp OR 'xenarthra'/exp OR 'haplorhini'/exp OR 'strepsirhini'/exp OR 'platyrrhini'/exp OR 'tarsii'/exp OR 'catarrhini'/exp OR 'cercopithecidae'/exp OR 'hylobatidae'/exp OR 'hominidae'/exp OR 'gorilla gorilla'/exp OR 'pan paniscus'/exp OR 'pan troglodyte' OR 'pongo pygmaeus'/exp OR animals OR animal OR mice OR mus OR mouse OR murine OR woodmouse OR rats OR rat OR murinae OR muridae OR cottonrat OR cottonrats OR hamster OR hamsters OR cricetinae OR rodentia OR rodent OR rodents OR pigs OR pig OR swine OR swines OR piglets OR piglet OR boar OR boars OR 'sus scrofa' OR ferrets OR ferret OR polecat OR polecats OR 'mustela putorius' OR 'guinea pigs' OR 'guinea pig' OR cavia OR callithrix OR marmoset OR marmosets OR cebuella OR hapale OR octodon OR chinchilla OR chinchillas OR gerbillinae OR gerbil OR gerbils OR jird OR jirds OR merione OR meriones OR rabbits OR rabbit OR hares OR hare OR diptera OR flies OR fly OR dipteral OR drosphila OR drosophilidae OR cats OR cat OR carus OR felis OR nematoda OR nematode OR nematodes OR sipunculida OR dogs OR dog OR canine OR canines OR canis OR sheep OR sheeps OR mouflon OR mouflons OR ovis OR goats OR goat OR capra OR capras OR rupicapra OR chamois OR haplorhini OR monkey OR monkeys OR anthropoidea OR anthropoids OR saguinus OR tamarin OR tamarins OR leontopithecus OR hominidae OR ape OR apes OR

pan OR paniscus OR 'pan paniscus' OR bonobo OR bonobos OR troglodytes OR 'pan troglodytes' OR gibbon OR gibbons OR siamang OR siamangs OR nomascus OR symphalangus OR chimpanzee OR chimpanzees OR prosimians OR 'bush baby' OR prosimian OR 'bush babies' OR galagos OR galago OR pongidae OR gorilla OR gorillas OR 'pongo pygmaeus' OR orangutans OR pygmaeus OR lemur OR lemurs OR lemuridae OR horse OR horses OR pongo OR equus OR cow OR calf OR bull OR chicken OR chickens OR gallus OR quail OR bird OR birds OR quails OR poultry OR poultries OR fowl OR fowls OR reptile OR reptilia OR reptiles OR snakes OR snake OR lizard OR lizards OR alligator OR alligators OR crocodile OR crocodiles OR turtle OR turtles OR amphibian OR amphibians OR amphibia OR frog OR frogs OR bombina OR salientia OR toad OR toads OR 'epidalea calamita' OR salamander OR salamanders OR eel OR eels OR fish OR fishes OR pisces OR catfish OR catfishes OR siluriformes OR arius OR heteropneustes OR sheatfish OR perch OR perches OR percidae OR perca OR trout OR trouts OR char OR chars OR salvelinus OR 'fathead minnow' OR minnow OR cyprinidae OR carps OR carp OR zebrafish OR zebrafishes OR goldfish OR goldfishes OR guppy OR guppies OR chub OR chubs OR tinca OR barbels OR barbus OR pimephales OR promelas OR 'poecilia reticulata' OR mullet OR mullets OR seahorse OR seahorses OR 'mugil curema' OR 'atlantic cod' OR shark OR sharks OR catshark OR anguilla OR salmonid OR salmonids OR whitefish OR whitefishes OR salmon OR salmons OR sole OR solea OR 'sea lamprey' OR lamprey OR lampreys OR pumpkinseed OR sunfish OR sunfishes OR tilapia OR tilapias OR turbot OR turbots OR flatfish OR flatfishes OR sciuridae OR squirrel OR squirrels OR chipmunk OR chipmunks OR suslik OR susliks OR vole OR voles OR lemming OR lemmings OR muskrat OR muskrats OR lemmus OR otter OR otters OR marten OR martens OR martes OR weasel OR badger OR badgers OR ermine OR mink OR sable OR sables OR gulo OR gulos OR wolverine OR wolverines OR minks OR mustela OR llama OR llamas OR alpaca OR alpacas OR camelid OR camelids OR guanaco OR guanacos OR chiroptera OR chiropteras OR bat OR bats OR fox OR foxes OR iguana OR iguanas OR 'xenopus laevis' OR parakeet OR parakeets OR parrot OR parrots OR donkey OR donkeys OR mule OR mules OR zebra OR zebras OR shrew OR shrews OR bison OR bisons OR buffalo OR buffaloes OR deer OR deers OR bear OR bears OR panda OR pandas OR 'wild hog' OR 'wild boar' OR fitchew OR fitch OR beaver OR beavers OR jerboa OR jerboas OR capybara OR capybaras)

Web of Science (502):

TOPIC: (vesicle OR vesicles OR microvesicle OR microvesicles OR micro-vesicle OR micro-vesicles OR nanovesicle OR nanovesicles OR nano-vesicle OR nano-vesicles OR microparticle OR microparticles OR micro-particle OR micro-particles OR exovesicle OR exovesicles OR “platelet dust” OR “platelet dusts” OR “apoptotic body” OR “apoptotic bodies” OR exosome OR exosomes OR secretome OR secretomes OR dexosome OR dexosomes OR texosome OR texosomes OR epididymosome OR epididymosomes OR tolerosome OR tolerosomes OR prostasome OR prostasomes OR ectosome OR ectosomes) AND TOPIC: ("myocardial infarction" OR "myocardial infarctions" OR "myocardial infarct" OR "myocardial infarcts" OR "cardiac infarction" OR "cardiac infarctions" OR "ventricular infarction" OR "ventricular infarctions" OR "myocardium infarction" OR "heart infarction" OR "heart infarctions" OR "myocardial ischemia" OR "myocardial ischemias" OR "myocardial necrosis" OR "myocardial necroses" OR "myocardial reperfusion" OR "myocardial reperfusions" OR "myocardial remodel" OR "myocardial remodeling" OR "ventricular remodeling" OR "atrial remodeling" OR "heart attack" OR "heart attacks" OR "coronary occlusion" OR "coronary occlusions" OR "coronary ligation" OR "coronary ligations") AND TOPIC: (animals OR animal OR mice OR mus OR mouse OR murine OR woodmouse OR rats OR rat OR murinae OR muridae OR cottonrat OR cottonrats OR hamster OR hamsters OR cricetinae OR rodentia OR rodent OR rodents OR pigs OR pig OR swine OR swines OR piglets OR piglet OR boar OR boars OR “sus scrofa” OR ferrets OR ferret OR polecat OR polecats OR “mustela putorius” OR “guinea pigs” OR “guinea pig” OR cavia OR callithrix OR marmoset OR marmosets OR cebuella OR hapale OR octodon OR chinchilla OR chinchillas OR gerbillinae OR gerbil OR gerbils OR jird OR jirds OR merione OR meriones OR rabbits OR rabbit OR hares OR hare OR diptera OR flies OR fly OR dipteral OR drosphila OR drosophilidae OR cats OR cat OR carus OR felis OR nematoda OR nematode OR nematodes OR sipunculida OR dogs OR dog OR canine OR canines OR canis OR sheep OR sheeps OR mouflon OR mouflons OR ovis OR goats OR goat OR capra OR capras OR rupicapra OR chamois OR haplorhini OR monkey OR monkeys OR anthropoidea OR anthropoids OR saguinus OR tamarin OR tamarins OR leontopithecus OR hominidae OR ape OR apes OR pan OR paniscus OR “pan paniscus” OR bonobo OR bonobos OR troglodytes OR “pan troglodytes” OR gibbon OR gibbons OR siamang OR siamangs OR nomascus OR symphalangus OR chimpanzee OR chimpanzees OR prosimians OR “bush baby” OR prosimian OR “bush babies” OR galagos OR galago OR pongidae OR gorilla OR gorillas OR “pongo pygmaeus” OR orangutans OR pygmaeus OR lemur OR lemurs OR lemuridae OR horse OR horses OR pongo OR equus OR cow OR calf OR bull OR chicken OR chickens OR gallus OR quail OR bird OR birds OR quails OR poultry OR poultries OR fowl OR fowls OR reptile OR reptilia OR reptiles OR snakes OR snake OR lizard OR lizards OR alligator OR alligators OR crocodile OR crocodiles OR turtle OR turtles OR amphibian OR amphibians OR amphibia OR frog OR frogs OR bombina OR salientia OR toad OR toads OR “epidalea calamita” OR salamander OR salamanders OR eel OR eels OR fish OR fishes OR pisces OR catfish OR catfishes OR siluriformes OR arius OR heteropneustes OR sheatfish OR perch OR perches OR percidae OR perca OR trout OR trouts OR char OR chars OR salvelinus OR “fathead minnow” OR minnow OR cyprinidae OR carps OR carp OR zebrafish OR zebrafishes OR goldfish OR goldfishes OR guppy OR guppies OR chub OR chubs OR tinca OR barbels OR barbus OR pimephales OR promelas OR “poecilia reticulata” OR mullet OR mullets OR seahorse OR seahorses OR “mugil curema” OR “atlantic cod” OR shark OR sharks OR catshark OR anguilla OR salmonid OR salmonids OR whitefish OR whitefishes OR salmon OR salmons OR sole OR solea OR “sea lamprey” OR lamprey OR lampreys OR pumpkinseed OR sunfish OR sunfishes OR tilapia OR tilapias OR

turbot OR turbots OR flatfish OR flatfishes OR sciuridae OR squirrel OR squirrels OR chipmunk OR chipmunks OR suslik OR susliks OR vole OR voles OR lemming OR lemmings OR muskrat OR muskrats OR lemmus OR otter OR otters OR marten OR martens OR martes OR weasel OR badger OR badgers OR ermine OR mink OR sable OR sables OR gulo OR gulos OR wolverine OR wolverines OR minks OR mustela OR llama OR llamas OR alpaca OR alpacas OR camelid OR camelids OR guanaco OR guanacos OR chiroptera OR chiropteras OR bat OR bats OR fox OR foxes OR iguana OR iguanas OR "xenopus laevis" OR parakeet OR parakeets OR parrot OR parrots OR donkey OR donkeys OR mule OR mules OR zebra OR zebras OR shrew OR shrews OR bison OR bisons OR buffalo OR buffaloes OR deer OR deers OR bear OR bears OR panda OR pandas OR "wild hog" OR "wild boar" OR fitchew OR fitch OR beaver OR beavers OR jerboa OR jerboas OR capybara OR capybaras)
Indexes=SCI-EXPANDED Timespan=All years

SUPPLEMENTARY REFERENCES

1. de Couto G, Gallet R, Cambier L, Jaghatspanyan E, Makkar N, Dawkins JF, Berman BP, Marbán E. Exosomal MicroRNA Transfer Into Macrophages Mediates Cellular Postconditioning. Circulation. 2017; 136:200–14. https://doi.org/10.1161/CIRCULATIONAHA.116.024590 PMID:28411247

2. Gallet R, Dawkins J, Valle J, Simsolo E, de Couto G, Middleton R, Tseliou E, Luthringer D, Kreke M, Smith RR, Marbán L, Ghaleb B, Marbán E. Exosomes secreted by cardiosphere-derived cells reduce scarring, attenuate adverse remodelling, and improve function in acute and chronic porcine myocardial infarction. Eur Heart J. 2017; 38:201–11. PMID:28158410

3. Ibrahim AG, Cheng K, Marbán E. Exosomes as critical agents of cardiac regeneration triggered by cell therapy. Stem Cell Reports. 2014; 2:606–19. https://doi.org/10.1016/j.stemcr.2014.04.006 PMID:24936449

4. Tseliou E, Fouad J, Reich H, Slipczuk L, de Couto G, Aminzadeh M, Middleton R, Valle J, Weixin L, Marbán E. Fibroblasts Rendered Antifibrotic, Antiapoptotic, and Angiogenic by Priming With Cardiosphere-Derived Extracellular Membrane Vesicles. J Am Coll Cardiol. 2015; 66:599–611. https://doi.org/10.1016/j.jacc.2015.05.068 PMID:26248985

5. Agarwal U, George A, Bhutani S, Ghosh-Choudhary S, Maxwell JT, Brown ME, Mehta Y, Platt MO, Liang Y, Sahoo S, Davis ME. Experimental, Systems, and Computational Approaches to Understanding the MicroRNA-Mediated Reparative Potential of Cardiac Progenitor Cell-Derived Exosomes From Pediatric Patients. Circ Res. 2017; 120:701–12. https://doi.org/10.1161/CIRCRESAHA.116.309935 PMID:27872050

6. Barile L, Lionetti V, Cervio E, Matteucci M, Gherghiceanu M, Popescu LM, Torre T, Siclari F, Moccetti T, Vassalli G. Extracellular vesicles from human cardiac progenitor cells inhibit cardiomyocyte apoptosis and improve cardiac function after myocardial infarction. Cardiovasc Res. 2014; 103:530–41. https://doi.org/10.1093/cvr/cvu167 PMID:25016614

7. Barile L, Cervio E, Lionetti V, Milano G, Ciullo A, Biemmi V, Bolis S, Altomare C, Matteucci M, Di Silvestre D, Brambilla F, Fertig TE, Torre T, et al. Cardioprotection by cardiac progenitor cell-secreted exosomes: role of pregnancy-associated plasma protein-A. Cardiovasc Res. 2018; 114:992–1005. https://doi.org/10.1093/cvr/cvy055 PMID:29518183

8. El Harane N, Kervadec A, Bellamy V, Pidial L, Neametalla HJ, Perier MC, Lima Correa B, Thiébault L, Cagnard N, Duché A, Brunaud C, Lemitre M, Gauthier J, et al. Acellular therapeutic approach for heart failure: in vitro production of extracellular vesicles from human cardiovascular progenitors. Eur Heart J. 2018; 39:1835–47. https://doi.org/10.1093/eurheartj/ehy012 PMID:29420830

9. Kervadec A, Bellamy V, El Harane N, Arakélian L, Vanneaux V, Cacciapuoti I, Nemetalla H, Périer MC, Toeg HD, Richart A, Lemitre M, Yin M, Loyer X, et al. Cardiovascular progenitor-derived extracellular vesicles recapitulate the beneficial effects of their parent cells in the treatment of chronic heart failure. J Heart Lung Transplant. 2016; 35:795–807. https://doi.org/10.1016/j.healun.2016.01.013 PMID:27041495

10. Chen CW, Wang LL, Zaman S, Gordon J, Arisi MF, Venkataraman CM, Chung JJ, Hung G, Gaffey AC, Spruce LA, Fazelinia H, Gorman RC, Seeholzer SH, et al. Sustained release of endothelial progenitor cell-derived extracellular vesicles from shear-thinning hydrogels improves angiogenesis and promotes function after myocardial infarction. Cardiovasc Res. 2018; 114:1029–40. https://doi.org/10.1093/cvr/cvy067 PMID:29566124

11. Khan M, Nickoloff E, Abramova T, Johnson J, Verma SK, Krishnamurthy P, Mackie AR, Vaughan E, Garikipati VN, Benedict C, Ramirez V, Lambers E, Ito A, et al. Embryonic stem cell-derived exosomes promote endogenous repair mechanisms and enhance cardiac function following myocardial infarction. Circ Res. 2015; 117:52–64. https://doi.org/10.1161/CIRCRESAHA.117.305990 PMID:25904597

12. Adamiak M, Cheng G, Bobis-Wozowicz S, Zhao L, Kedracka-Krok S, Samanta A, Karnas E, Xuan YT, Skupien-Rabian B, Chen X, Jankowska U, Girgis M, Sekula M, et al. Induced Pluripotent Stem Cell (iPSC)-Derived Extracellular Vesicles Are Safer and More Effective for Cardiac Repair Than iPSCs. Circ Res. 2018; 122:296–309. https://doi.org/10.1161/CIRCRESAHA.117.311769 PMID:29118058

13. Arslan F, Lai RC, Smeets MB, Akeroyd L, Choo A, Aguor EN, Timmers L, van Rijen HV, Doevendans PA, Pasterkamp G, Lim SK, de Kleijn DP. Mesenchymal stem cell-derived exosomes increase ATP levels, decrease oxidative stress and activate PI3K/Akt

pathway to enhance myocardial viability and prevent adverse remodeling after myocardial ischemia/ reperfusion injury. Stem Cell Res (Amst). 2013; 10:301–12. https://doi.org/10.1016/j.scr.2013.01.002 PMID:23399448

14. Bian S, Zhang L, Duan L, Wang X, Min Y, Yu H. Extracellular vesicles derived from human bone marrow mesenchymal stem cells promote angiogenesis in a rat myocardial infarction model. J Mol Med (Berl). 2014; 92:387–97. https://doi.org/10.1007/s00109-013-1110-5 PMID:24337504

15. Cui X, He Z, Liang Z, Chen Z, Wang H, Zhang J. Exosomes From Adipose-derived Mesenchymal Stem Cells Protect the Myocardium Against Ischemia/ Reperfusion Injury Through Wnt/β-Catenin Signaling Pathway. J Cardiovasc Pharmacol. 2017; 70:225–31. https://doi.org/10.1097/FJC.0000000000000507 PMID:28582278

16. Lai RC, Arslan F, Tan SS, Tan B, Choo A, Lee MM, Chen TS, Teh BJ, Eng JK, Sidik H, Tanavde V, Hwang WS, Lee CN, et al. Derivation and characterization of human fetal MSCs: an alternative cell source for large-scale production of cardioprotective microparticles. J Mol Cell Cardiol. 2010; 48:1215–24. https://doi.org/10.1016/j.yjmcc.2009.12.021 PMID:20064522

17. Lai RC, Arslan F, Lee MM, Sze NS, Choo A, Chen TS, Salto-Tellez M, Timmers L, Lee CN, El Oakley RM, Pasterkamp G, de Kleijn DP, Lim SK. Exosome secreted by MSC reduces myocardial ischemia/reperfusion injury. Stem Cell Res (Amst). 2010; 4:214–22. https://doi.org/10.1016/j.scr.2009.12.003 PMID:20138817

18. Shao L, Zhang Y, Lan B, Wang J, Zhang Z, Zhang L, Xiao P, Meng Q, Geng YJ, Yu XY, Li Y. MiRNA-Sequence Indicates That Mesenchymal Stem Cells and Exosomes Have Similar Mechanism to Enhance Cardiac Repair. BioMed Res Int. 2017; 2017:4150705. https://doi.org/10.1155/2017/4150705 PMID:28203568

19. Teng X, Chen L, Chen W, Yang J, Yang Z, Shen Z. Mesenchymal Stem Cell-Derived Exosomes Improve the Microenvironment of Infarcted Myocardium Contributing to Angiogenesis and Anti-Inflammation. Cell Physiol Biochem. 2015; 37:2415–24. https://doi.org/10.1159/000438594 PMID:26646808

20. Timmers L, Lim SK, Arslan F, Armstrong JS, Hoefer IE, Doevendans PA, Piek JJ, El Oakley RM, Choo A, Lee CN, Pasterkamp G, de Kleijn DP. Reduction of myocardial infarct size by human mesenchymal stem cell conditioned medium. Stem Cell Res (Amst). 2007; 1:129–37. https://doi.org/10.1016/j.scr.2008.02.002 PMID:19383393

21. Wang N, Chen C, Yang D, Liao Q, Luo H, Wang X, Zhou F, Yang X, Yang J, Zeng C, Wang WE. Mesenchymal stem cells-derived extracellular vesicles, via miR-210, improve infarcted cardiac function by promotion of angiogenesis. Biochim Biophys Acta Mol Basis Dis. 2017; 1863:2085-2092. https://doi.org/10.1016/j.bbadis.2017.02.023 PMID:28249798

22. Yu B, Kim HW, Gong M, Wang J, Millard RW, Wang Y, Ashraf M, Xu M. Exosomes secreted from GATA-4 overexpressing mesenchymal stem cells serve as a reservoir of anti-apoptotic microRNAs for cardioprotection. Int J Cardiol. 2015; 182:349–60. https://doi.org/10.1016/j.ijcard.2014.12.043 PMID:25590961

23. Zhao Y, Sun X, Cao W, Ma J, Sun L, Qian H, Zhu W, Xu W. Exosomes Derived from Human Umbilical Cord Mesenchymal Stem Cells Relieve Acute Myocardial Ischemic Injury. Stem Cells Int. 2015; 2015:761643. https://doi.org/10.1155/2015/761643 PMID:26106430

24. Zhu J, Lu K, Zhang N, Zhao Y, Ma Q, Shen J, Lin Y, Xiang P, Tang Y, Hu X, Chen J, Zhu W, Webster KA, et al. Myocardial reparative functions of exosomes from mesenchymal stem cells are enhanced by hypoxia treatment of the cells via transferring microRNA-210 in an nSMase2-dependent way. Artif Cells Nanomed Biotechnol. 2018; 46:1659–70. PMID:29141446

25. Gould SJ, Raposo G. As we wait: coping with an imperfect nomenclature for extracellular vesicles. J Extracell Vesicles. 2013; 2:20389. https://doi.org/10.3402/jev.v2i0.20389 PMID:24009890

26. Haraszti RA, Didiot MC, Sapp E, Leszyk J, Shaffer SA, Rockwell HE, Gao F, Narain NR, DiFiglia M, Kiebish MA, Aronin N, Khvorova A. High-resolution proteomic and lipidomic analysis of exosomes and microvesicles from different cell sources. J Extracell Vesicles. 2016; 5:32570. https://doi.org/10.3402/jev.v5.32570 PMID:27863537

27. Crescitelli R, Lässer C, Szabó TG, Kittel A, Eldh M, Dianzani I, Buzás EI, Lötvall J. Distinct RNA profiles in subpopulations of extracellular vesicles: apoptotic bodies, microvesicles and exosomes. J Extracell Vesicles. 2013; 2:20677. https://doi.org/10.3402/jev.v2i0.20677 PMID:24223256

28. Mateescu B, Kowal EJ, van Balkom BW, Bartel S, Bhattacharyya SN, Buzás EI, Buck AH, de Candia P, Chow FW, Das S, Driedonks TA, Fernández-Messina L, Haderk F, et al. Obstacles and opportunities in the functional analysis of extracellular vesicle RNA - an ISEV position paper. J Extracell Vesicles. 2017; 6:1286095.
https://doi.org/10.1080/20013078.2017.1286095
PMID:28326170

29. Hooijmans CR, Tillema A, Leenaars M, Ritskes-Hoitinga M. Enhancing search efficiency by means of a search filter for finding all studies on animal experimentation in PubMed. Lab Anim. 2010; 44:170–75.
https://doi.org/10.1258/la.2010.009117
PMID:20551243
